# The role of androgen receptor activity mediated by the CAG repeat polymorphism in the pathogenesis of PCOS

**Published:** 2013-03-25

**Authors:** N Baculescu

**Affiliations:** “Carol Davila" University of Medicine and Pharmacy, Bucharest, Romania

**Keywords:** CAG repeat polymorphism, AR, PCOS

## Abstract

Polycystic ovary syndrome (PCOS), one of the most common and complex endocrine disorders affecting up to 15 % of reproductive age women, is considered a predominantly hyperandrogenic syndrome according to the Androgen Excess Society. It is generally accepted that androgens determine the characteristic features of PCOS; in this context, a hyperactive androgen receptor (AR) at the levels of the GnRH pulse generator in the hypothalamus and at the granulosa cells in the ovary, skeletal muscle or adipocytes senses initially normal testosterone and dihydrotestosterone as biochemical hyperandrogenism and might be a crucial connection between the vicious circles of the PCOS pathogenesis.

Polymorphism of the AR gene has been associated with different androgen pattern diseases. Several studies have demonstrated an association between AR with increased activity encoded by shorter CAG repeat polymorphism in the exon 1 of the AR gene and PCOS, although there are conflicting results in this field. The phenomenon is more complex because the AR activity is determined by the epigenetic effect of X chromosome inactivation (XCI). Moreover, we must evaluate the AR as a dynamic heterocomplex, with a large number of coactivators and corepressors that are essential to its function, thus mediating tissue-specific effects. In theory, any of these factors could modify the activity of AR, which likely explains the inconsistent results obtained when this activity was quantified by only the CAG polymorphism in PCOS.

## Background

Polycystic ovary syndrome (PCOS) is a complex endocrine disorder [**1-3**] that affects up to 15% of reproductive age women [**4**]. Although the aetiology remains unknown, some investigators consider that androgen excess is the sine qua non of PCOS [**5**], which is consistent with the theory that androgens determine the characteristic features of the syndrome, for example, oligo-anovulation and infertility, inhibition of follicle development with polyfollicular ovarian morphology, hirsutism, acne or androgenic alopecia [**6-10**]. 

 Experiments conducted in animal models as well as equivalent natural situations in humans, i.e., pregnancy in PCOS or congenital adrenal hyperplasia, demonstrated that exposure to androgen excess during the foetal life and infancy determines the PCOS features in adult life, with LH hypersecretion, oligomenorrhoea, polyfollicular enlarged ovaries, insulin resistance, abdominal obesity, impaired insulin response to glucose and dyslipidemia [**11-17**]. These data provide evidence that androgen excess is a key factor in the PCOS etiopathogeny. 

 In women with PCOS, the abnormality in the regulation of hypothalamic GnRH secretion, with persistently rapid GnRH pulsatility, pituitary synthesis of LH over that of FSH, and increased LH concentrations and LH/FSH ratios, has been explained, at least in part, by a decreased sensitivity of the GnRH pulse generator to the progesterone suppression [**18-20**]. In these adult women with PCOS, the blockade of androgen action by flutamide administration restored the sensitivity of GnRH to estradiol and progesterone, thus supporting the role of an increased androgenic activity mediated by the androgen receptor (AR) to reduce the progesterone negative feedback at the level of the GnRH pulse generator [**18,21**]. More specifically, experiments conducted in animal models showed that a special KNDy subpopulation of cells in the arcuate nucleus, which colocalise three important neuropeptides, kisspeptin, neurokinin b and dynorphin, express steroid receptors that include AR and connect with GnRH neurons in a network, are responsible for transporting the testosterone influence on GnRH neurons in the hypothalamic GnRH pulse generator [**22**]. 

 Although the insulin resistance of PCOS partially has a genetic substrate [**23,24**], an increased androgenic activity could amplify insulin resistance as soon as testosterone in physiological concentrations enhanced the insulin-induced IRS-1 serine 636/639 phosphorylation in differentiated rat skeletal muscle myotubes [**25**]. AR is present in human addipose tissue, at the level of preadypocites and adipocytes [**26,27**] and Corbould demonstrated that testosterone induces a selective insulin resistance in cultured subcutaneous adipocytes of women, with action mediated by the classical AR, given that AR antagonists, cyproterone acetate and flutamide attenuated the effect of testosterone on the glucose uptake [**28**]. 

 These data suggest a link between an increased androgenic activity, AR mediated, and the development of insulin resistance in PCOS. 

 Whereas the classic target tissues such as muscle and fat manifest insulin resistance in PCOS, the ovaries of these patients remain sensitive to insulin or perhaps hypersensitive to it [**29**]. In this condition, compensatory hyperinsulinaemia in synergistic action with LH will amplify all steroidogenesis and specifically testosterone synthesis at the level of responsive ovarian theca cells. The androgens, either circulatory or produced locally, impair the ability of primary follicles to grow normally, with a marked increase in the number of growing preantral and antral follicles, particularly in the population of growing and classic primary follicles, leading to the typical polycystic ovarian morphology [**30**]. Several studies have demonstrated that high-dose androgen treatment of transsexual women induced enlarged ovaries with increased numbers of cystic follicles and theca-interstitial hyperplasia, which meet the morphological criteria for PCOS [**31-33**]. Although the gonadotropins were suppressed in these subjects, their ovaries were not suppressed. These observations suggested that androgens per se might induce ovarian follicular and theca interstitial growth [**7**]. These data, correlated with experiments in non-human primates [**7**] and the evidence for expression of AR in the human ovary, at germinal epithelium, granulosa cells of antral follicles, corpus luteum, and thecal and stromal cells [**34**], indicated that the characteristic polycystic ovarian morphology, with enlarged ovaries and numerous small follicular cysts, might be the result of direct, receptor-mediated androgen activity. 

**Fig. 1:**
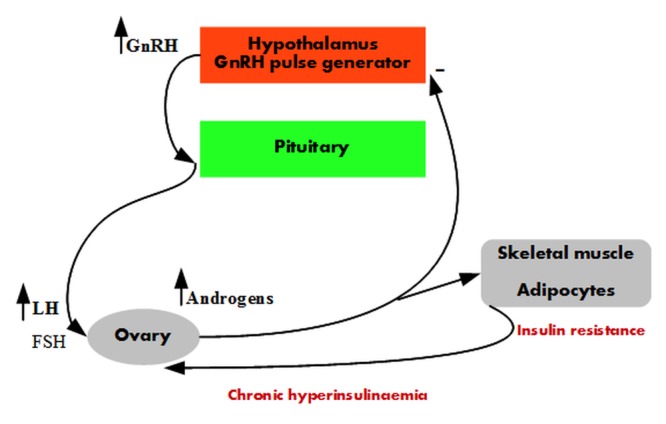
Increased androgenic activity in the PCOS pathogenesis. There is increased GnRH pulse frequency with preferential LH secretion at the pituitary level. LH stimulates testosterone synthesis at the level of theca cells in the ovary. Testosterone reduces the negative progesterone feedback on the GnRH pulse generator and amplifies insulin resistance in the adipocytes and skeletal muscle. Compensatory hyperinsulinaemia in synergy with LH amplifies the testosterone production in the ovary.

 Therefore, the androgens modulate different axes in the PCOS pathogenesis (**Fig. 1**), with androgenic effects mediated by the AR.

Although the PCOS has demonstrated a familial clustering and a suspected genetic basis, to date, there is no evidence to support that a single gene defect might be responsible for this syndrome [**1,10,35**]. However, several studies have supported AR with higher activity, mediated by AR gene polymorphism, as a significant determining factor in the PCOS development [**10,36-38**] via increasing androgenic activity, although androgens are in the normal range. 

 Molecular basis of AR activity and general consequences in androgen pattern diseases

**Fig. 2:**
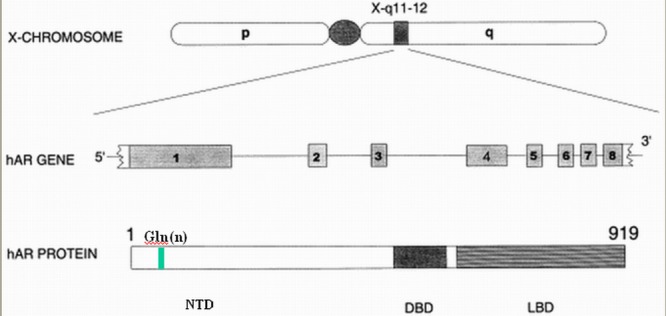
The AR gene and encoded AR protein The AR gene consists of eight exons, encoding three domains in the AR protein: the transcription regulation domain (NTD), the DNA-binding domain (DBD) and the specific ligand-binding domain (LBD). Exon 1 contains the polymorphic CAG repeat, which encodes a polyglutamine (Gln) n tract in NTD. Elsevier granted permission to reproduce and modify this figure.

 The AR, encoded by the AR gene located at Xq11-q12, is a nuclear transcription factor and member of the steroid receptor superfamily [**39**]. Its structure is common to all of the nuclear receptors, comprising an N-terminal activation domain, encoded by the exon 1 of the gene, a central DNA-binding domain (DBD), encoded by the exons 2 and 3, and a C-terminal ligand-binding domain (LBD) encoded by the exons 4-8 [**40,41**] (**Fig. 2**). 

The unbound AR is inactive in the cytoplasm as a large dynamic heterocomplex, together with heat shock proteins (Hsp70 and Hsp90) and their co-chaperones [**42**]. After ligand binding, AR adopts an active conformation that facilitates the dissociation of heat shock proteins, dimerisation, and binding to response elements in the promoters of responsive genes in the nucleus. The N-terminal activation domain accounts for more than 60% of the AR protein and has a potent transcriptional activator function (AF-1) at the level of androgen response elements [**43**]. 

 So far, have been described different point mutations, deletions or insertions at the level of the AR gene that influence the activity of AR in different diseases, such as complete androgen insensitivity syndrome (CAIS) [**44**], partial androgen insensitivity syndrome (Reifenstein syndrome) [**45**] or prostate cancer [**44**]. 

 Moreover, has been shown that the activity of AR is influenced by a genetic polymorphism in exon 1 of the AR gene. There are two polymorphic trinucleotide repeat fragments, CAGn and GGNn, in the first exon of the AR gene, encoding two polyglutamine and polyglycine tracts in the N-terminal activation domain. Although the functional consequences of variation in the length of the GGNn fragment are unclear, several in vitro studies have evidenced an inverse relationship between the length of the CAG polymorphic repeat (normal range, 8-35) and the AR transcriptional activity [**46-49**]; however, there is no uniform consensus [**50**]. 

 In vivo studies support the majority of functional data, which demonstrate a negative correlation between the CAG repeat number and AR activity. Healthy men whose AR has a CAG repeat length of greater than 28 have an increased incidence of impaired spermatogenesis and infertility [**47**], whereas the expansion of the CAG repeat length of over 40 is related to SBMA, a rare neuromuscular disorder characterised by spinal and bulbar muscular atrophy and is associated with androgen insensitivity, decreased virilisation, testicular atrophy, reduced sperm production, and infertility [**51**]. Expanded CAGn repeat in the AR gene modulates other male-specific phenotypes, such as prostate growth under androgen substitution in hypogonadal men [**52**] or different phenotypic features in untreated patients with Klinefelter syndrome [**53,54**]. 

 Conversely, shorter AR polyglutamine tracts and thus a more transcriptionally active AR have been associated with prostate cancer [**55-59**] or premature pubarche [**60**]. 

CAG polymorphism of AR gene in PCOS

 According to functional and in vivo data, CAG repeat polymorphism, encoding an AR with increased activity, might play a role in PCOS pathogenesis. However, several studies centred on the association between the CAG repeat polymorphism of the AR gene and PCOS have conflicting results [**10,36-38,61-69**]. 

 Some authors observed that short androgen receptor CAGn alleles tended to appear more frequently in women with PCOS than in controls [**36**]; this finding is consistent with that of another study that described all of the subjects with 15 or fewer CAG repeats as part of the PCOS group [**37**]. Azziz et al and Schuring et al reported significantly lower CAG repeats in patients with PCOS than in controls [**10,38,69**], a finding consistent with our preliminary data that demonstrated significantly shorter biallelic means of CAG repeats in patients with PCOS than in normal women [**67**]. 

 Mohlig et al demonstrated that in women with PCOS, the AR CAG repeat polymorphism has metabolic consequences by modifying the impact of testosterone on insulin resistance, which is consistent with prior known functional data. In a statistical model that described 42.5% of the HOMA-IR variation, the elevated testosterone increased the insulin resistance in carriers of short CAG lengths; this effect attenuated with increasing biallelic CAG length until it turned into the opposite at a CAG length of longer than 23 [**70**]. 

 Other studies showed no difference in CAG repeat length between patients with PCOS and controls, regardless of the fact that both alleles were considered together or separately [**61,63-66,68**][61,63-66,68]; however, when compared PCOS groups constructed by androgen levels, one of these studies showed a trend for lower CAG biallelic means in the subgroup of women with PCOS and lower testosterone levels, which was consistent with a role for androgen hyperactivity in the development of the syndrome [**61**]. 

 Contrary to the theory of an inverse relation between CAG repeat number and receptor activity, the study published by Hickey et al has shown that infertile women with PCOS exhibited a greater frequency of CAG alleles or biallelic means greater than 22 repeats compared with both the fertile controls and the general population [**62**]. 

 Regarding clinical hyperandrogenism of PCOS, several studies found that neither hirsutism (or modified Ferriman-Gallwey score) [**38,61,65,68**] nor acne [**68**] was significantly associated with the CAG repeat polymorphism, whereas other studies found that short CAG repeat was associated with hirsutism and acne [**69-71**]. 

 In addition to the conflicting data on the association between genetically encoded CAG repeats and PCOS, we should consider the X chromosome inactivation (XCI) as a significant phenomenon present in females because the AR gene is located on the X chromosome; therefore, the AR activity is determined not only by the AR genotype but also by the epigenetic effect of XCI [**69**]. DNA methylation is an important epigenetic mechanism in X chromosome inactivation [**72**], which normally occurs in early embryogenesis as a random event. A nonrandom X inactivation, either due to environmental exposures or allelic differences, may contribute significantly to the expression of PCOS [**38**]. Experimentally induced animal models, in monkeys or sheep, showed that in utero hyperandrogenism exposure may lead to abnormalities of epigenetic reprogramming in foetal reproductive and metabolically active tissues and may produce an impact on the neuroendocrine development, gonadal differentiation and pancreatic organogenesis [**16**], which ultimately results in a PCOS phenotype in the adult life [**3,16**]. Prenatally androgenised female rhesus monkeys developed a PCOS-like phenotype as adults with increased visceral adipose tissue and basal serum insulin levels [**73**], conditions that correlated with different DNA methylation patterns in both infant and adult visceral adipose tissue [**74**]. An equivalent, naturally occurring situation with foetal exposure to the androgen excess occurs in pregnant women with PCOS [**13,16,75-77**]. 

 The transitory foetal hyperandrogenemic environment could modify (in humans as well as monkeys) the foetal DNA methylation patterns in various tissues, including the X chromosome inactivation pattern and AR expression in the active X chromosome, of which both are critical mechanisms involved in the activity of AR. 

 We hypothesise that a hyperandrogenemic environment could modify the process of methylation, including the AR expression in the active X chromosome, even when it appears in adult life, based on the data that demonstrated a loss of methylation of the LH receptor in the ovary of a mouse model with PCOS induced by the administration of dehydroepiandrosterone [**78**]. 

 However, the controversies have also persisted in the studies that analyse the XCI in patients with PCOS. (**Table 1**). 

**Table 1:** Studies of the CAG repeats and XCI in patients with PCOS

Article	Country	Ethnicity	No. of subjects PCOS	Controls	PCOS diagnostic criteria	CAGn genetically encoded	Conclusions XCI
Hickey 2002	Australia	Caucasian	122	83	NIH 1990 (infertile patients)	- greater frequency of CAG alleles or biallelic means >22 repeats in infertile patients with PCOS vs fertile controls or general population	- preferential expression of longer CAG repeats in PCOS
Shah 2008	USA	Caucasian and Black	270	165	NIH 1990	- a smaller biallelic mean of CAG repeats associated with increased odds of PCOS	- shorter CAG alleles, preferentially active in the PCOS with nonrandom X inactivation
Dasgupta 2010	India	Asian	249	296	Rotterdam 2003	- the mean CAG repeats number was similar between patients with PCOS and controls	- among the PCOS with non-random X- inactivation, alleles with <19 repeats were more frequently activated among patients than controls
Laisk 2010	Estonia	Caucasian	32	79	Rotterdam 2003	- no direct associations between AR CAG repeats and PCOS	- no direct associations between XCI and PCOS
Radian 2010	Romania	Caucasian	137	130	Rotterdam 2003	- biallelic means of CAG repeats were significantly shorter between patients with PCOS and controls	- the non-random subgroup of patients with PCOS has significantly shorter X-weighted biallelic means
Schuring 2011	Germany	Caucasian	72	179	Rotterdam 2003	- biallelic means of CAG repeats, significantly shorter between patients with PCOS and controls	- X-weighted biallelic means of CAG repeats were significantly shorter between patients with PCOS and controls

 There are several limitations of the CAG repeat polymorphism association studies in PCOS.

 1. Regarding genetically encoded CAG repeat number

 a. The inconsistent results could be explained, at least in part, by the different ethnic backgrounds of individuals with PCOS and controls, i.e., the Caucasian population of an immigration country [**62**] or the heterogenic populations of Chinese and Indians in Singapore [**10-61**]. The normal range of CAG repeats varied according to ethnicity (approximately 10–30 in Caucasians [**79**], slightly shorter in African-Americans and longer in Asians [**80-81**] and became important if the control subjects were recruited in a different population compared to the corresponding groups with PCOS [**37,61-63**].

 b. Other confounding factors could be the different criteria used to define PCOS or sample sizes of patients and controls. 

 2. Regarding the epigenetics

 a. Presently, only two HpaII methylation pattern sites have been assessed in the analysis of the AR gene by PCR-based assays after digestion of DNA with methylation-sensitive HpaII [**10,38,62,65-67**]. The results concluded that overall XCI was not significantly different between patients with PCOS and controls. However, three important studies have found that the shorter allele of the AR was preferentially active in PCOS in the subgroup of women with non-random XCI [**10,38,65**]. Our preliminary results demonstrated significantly lower biallelic means and X-weighted biallelic means of CAG repeat in the non-random subgroup of patients with PCOS [**67**].

 In the hirsute PCOS, one study concluded that normoandrogenemic hirsutism was associated with preferential methylation of the longer allele of the two AR alleles, allowing for the shorter (and presumably, more functional) allele to be expressed on the active X chromosome [**82**]. On the contrary, no difference in the X inactivation pattern was shown in other studies when the hirsutism was analysed in patients with PCOS [**10,38,83**]; however, in one of these, a discrete preferential expression of the shorter CAG allele was observed [**38**]. 

 In this context, supplementary information can be derived by investigating the methylation pattern of AR’s entire sites near the polymorphic CAG repeats because all of those CpG sites may be involved in regulating AR expression in the active X chromosome [**78**]. 

 b. We should consider that XCI can vary in different tissues. In all of the previous studies, XCI was generally tested by using blood samples, which does not appear to reflect the condition in androgen target tissues [**69**]. One important study demonstrated that total methylation of peripheral blood leukocyte DNA is unaltered in patients with PCOS vs. matched controls [**84**]. 

3. Finally, a large number of AR coactivators and corepressors have been identified as critical to the AR function. When they mediate tissue-specific androgen effects, a variation in the expression or function of these coregulators might contribute to the development of PCOS [**38,85**]. Future genetic and physiologic studies are required to identify the functional interactions between the CAG repeats and these AR coregulators [**38**]. 

## Conclusions

Genetic data that demonstrate a hyperactive androgen receptor in PCOS are limited to the evaluation of the CAG repeat polymorphism of the AR gene and are inconsistent, thereby requiring more in vitro studies to identify functional AR polymorphisms that affect AR transactivity and different PCOS phenotypes. The important role that AR with increased activity plays in the PCOS pathogenesis is interesting in light of continuous efforts to understand this complex disease, which might represent an important connection between the multiple characteristic pathways, including the androgens, neuroendocrine and insulin axis. 

## References

[1]  Escobar-Morreale HF, Luque-Ramirez M (2005). The molecular-genetic basis of functional hyperandrogenism and the polycystic ovary syndrome. Endocrine Reviews.

[2] Diamanti-Kandarakis E, Piperi  C (2005). Genetics of polycystic ovary syndrome: searching for the way out of the labyrinth. Human Reproduction Update.

[3] Li  ZX, Huang  HF (2008). Epigenetic abnormality: A possible mechanism underlying the fetal origin of polycystic ovary syndrome. Medical Hypotheses.

[4] Fauser  BCJM, Tarlatzis  BC (2012). Consensus on women's health aspects of polycystic ovary syndrome (PCOS). Human Reproduction.

[5] Goodarzi  MO, Dumesic  DA (2011). Polycystic ovary syndrome: etiology, pathogenesis and diagnosis. Nature Reviews Endocrinology.

[6] Hillier  SG, Tetsuka  M (1997). Role of androgens in follicle maturation and atresia. Baillieres Clinical Obstetrics and Gynaecology.

[7] Vendola  KA, Zhou  J (1998). Androgens stimulate early stages of follicular growth in the primate ovary. Journal of Clinical Investigation.

[8] Azziz  R, Woods  KS (2004). The prevalence and features of the polycystic ovary syndrome in an unselected population. Journal of Clinical Endocrinology & Metabolism.

[9] Dewailly  D, Catteau-Jonard S (2006). Oligoanovulation with polycystic ovaries but not overt hyperandrogenism. Journal of Clinical Endocrinology & Metabolism.

[10] Schuring  AN, Welp  A (2012). Role of the CAG Repeat Polymorphism of the Androgen Receptor Gene in Polycystic Ovary Syndrome (PCOS). Experimental and Clinical Endocrinology & Diabetes.

[11] Abbott  (2007). Fetal programming of polycystic ovary syndrome. In: Kovacs G. and Norman R, eds. Polycystic ovary syndrome.

[12] Xita  N, Tsatsoulis  A (2006). Review: Fetal programming of polycystic ovary syndrome by androgen excess: Evidence from experimental, clinical, and genetic association studies. Journal of Clinical Endocrinology & Metabolism.

[13]  Sir-Petermann T, Maliqueo  M (2002). Maternal serum androgens in pregnant women with polycystic ovarian syndrome: possible implications in prenatal androgenization. Human Reproduction.

[14]  McClamrock HD, Adashi  EY (1992). Gestational hyperandrogenism. Fertil Steril.

[15] Barnes  RB, Rosenfield  RL (1994). Ovarian hyperandrogenism as a result of congenital adrenal virilizing disorders: evidence for perinatal masculinization of neuroendocrine function in women. J Clin Endocrinol Metab.

[16] Abbout  DH, Barnett  DK (2005). Androgen excess fetal programming of female reproduction: a developmental aetiology for polycystic ovary syndrome?. Human Reproduction Update.

[17] Hague  WM, Adams  J (1990). The prevalence of polycystic ovaries in patients with congenital adrenal hyperplasia and their close relatives. Clin Endocrinol.

[18] Blank  SK, McCartney  CR (2006). The origins and sequelae of abnormal neuroendocrine function in polycystic ovary syndrome. Human Reproduction Update.

[19] Pastor  CL, Griffin-Korf  ML (1998). Polycystic ovary syndrome: Evidence for reduced sensitivity of the gonadotropin-releasing hormone pulse generator to inhibition by estradiol and progesterone. Journal of Clinical Endocrinology & Metabolism.

[20] Blank  SK, McCartney  CR (2009). Modulation of Gonadotropin-Releasing Hormone Pulse Generator Sensitivity to Progesterone Inhibition in Hyperandrogenic Adolescent Girls-Implications for Regulation of Pubertal Maturation. Journal of Clinical Endocrinology & Metabolism.

[21] Eagleson  CA, Gingrich  MB (2000). Polycystic ovarian syndrome: Evidence that flutamide restores sensitivity of the gonadotropin-releasing hormone pulse generator to inhibition by estradiol and progesterone. Journal of Clinical Endocrinology & Metabolism.

[22] Lehman  MN, Coolen  LM (2010). Minireview: Kisspeptin/Neurokinin B/Dynorphin (KNDy) Cells of the Arcuate Nucleus: A Central Node in the Control of Gonadotropin-Releasing Hormone Secretion. Endocrinology.

[23]  El Mkadem SA, Lautier  C (2001). Role of allelic variants Gly972Arg of IRS-1 and Gly1057Asp of IRS-2 in moderate-to-severe insulin resistance of women with polycystic ovary syndrome. Diabetes.

[24] Hanzu  FA, Radian  S (2010). Association of insulin receptor genetic variants with polycystic ovary syndrome in a population of women from Central Europe. Fertility and Sterility.

[25] Allemand  MC, Irving  BA (2009). Effect of Testosterone on Insulin Stimulated IRS1 Ser Phosphorylation in Primary Rat Myotubes-A Potential Model for PCOS-Related Insulin Resistance. Plos One.

[26] Pedersen  SB, Fuglsig  S (1996). Identification of steroid receptors in human adipose tissue. European Journal of Clinical Investigation.

[27] Dieudonne  MN, Pecquery  R (1998). Androgen receptors in human preadipocytes and adipocytes: regional specificities and regulation by sex steroids. American Journal of Physiology-Cell Physiology.

[28] Corbould  A (2007). Chronic testosterone treatment induces selective insulin resistance in subcutaneous adipocytes of women. Journal of Endocrinology.

[29] Baillargeon  JP, Nestler  JE (2006). Commentary: Polycystic ovary syndrome: A syndrome of ovarian hypersensitivity to insulin?. Journal of Clinical Endocrinology & Metabolism.

[30] Maciel  GAR, Baracat  EC (2004). Stockpiling of transitional and classic primary follicles in ovaries of women with polycystic ovary syndrome. Journal of Clinical Endocrinology & Metabolism.

[31] Futterweit  W, Deligdisch  L (1986). Histopathological effects of exogenously administered testosterone in 19 female to male transsexuals. J Clin Endocrinol Metab.

[32] Pache  TD, Chadha  S (1991). Ovarian morphology in long-term androgen-treated female-to-male transsexuals. A human model for the study of PCOS?. Histopathology.

[33] Spinder  T, Spijkstra  JJ (1989). The effects of long term testosterone administration on pulsatile luteinizing hormone secretion and on ovarian histology in eugonadal female to male transsexual subjects. J Clin Endocr Metab.

[34] Chadha  S, Pache  TD (1994). Androgen Receptor Expression in Human Ovarian and Uterine Tissue of Long-Term Androgen-Treated Transsexual Women. Human Pathology.

[35] Franks  S, McCarthy  MI (2006). Development of polycystic ovary syndrome: involvement of genetic and environmental factors. International Journal of Andrology.

[36] Xita  N, Georgiou  I (2008). The role of sex hormone-binding globulin and androgen receptor gene variants in the development of polycystic ovary syndrome. Human Reproduction.

[37] Jaaskelainen  J, Korhonen  S (2005). Androgen receptor gene CAG length polymorphism in women with polycystic ovary syndrome. Fertility and Sterility.

[38] Shah  NA, Antoine  HJ (2008). Association of androgen receptor CAG repeat polymorphism and polycystic ovary syndrome. Journal of Clinical Endocrinology & Metabolism.

[39] Chang AR (1988). structure. Experimental and Clinical Endocrinol Diabetes.

[40] Beato  M, Herrlich  P (1995). Steroid-Hormone Receptors - Many Actors in Search of A Plot. Cell.

[41] Brinkmann  AO (2001). Molecular basis of androgen insensitivity. Molecular and Cellular Endocrinology.

[42] Pratt  WB, Toft  DO (1997). Steroid receptor interactions with heat shock protein and immunophilin chaperones. Endocrine Reviews.

[43] Dehm  SM, Tindall  DJ (2007). Androgen receptor structural and functional elements: Role and regulation in prostate cancer. Molecular Endocrinology.

[44] Newmark  JR (1992). Androgen receptor gene mutations in human prostate cancer. Proc Natl Acad Sci.

[45] Kohler  B, Lumbroso  S (2005). Androgen insensitivity syndrome: Somatic mosaicism of the androgen receptor in seven families and consequences for sex assignment and genetic counseling. Journal of Clinical Endocrinology & Metabolism.

[46] Chamberlain  NL, Driver  ED (1994). The Length and Location of Cag Trinucleotide Repeats in the Androgen Receptor N-Terminal Domain Affect Transactivation Function. Nucleic Acids Research.

[47] Tut  TG, Ghadessy  FJ (1997). Long polyglutamine tracts in the androgen receptor are associated with reduced trans-activation, impaired sperm production, and male infertility. Journal of Clinical Endocrinology & Metabolism.

[48] Beilin  J, Ball  EMA (2000). Effect of the androgen receptor CAG repeat polymorphism on transcriptional activity: specificity in prostate and non-prostate cell lines. Journal of Molecular Endocrinology.

[49] Choong  CS, Kemppainen  JA (1996). Reduced androgen receptor gene expression with first exon CAG repeat expansion. Molecular Endocrinology.

[50] Nenonen (2010). CAG repeat number is not inversely associated with androgen receptor activity in vitro. Molecular Human Reproduction.

[51] Fischbeck  KH, Lieberman  A (1999). Androgen receptor mutation in Kennedy's disease. Philosophical Transactions of the Royal Society of London Series B-Biological Sciences.

[52] Zitzmann  M, Depenbusch  M (2003). Prostate volume and growth in testosterone-substituted hypogonadal men are dependent on the CAG repeat polymorphism of the androgen receptor gene: A longitudinal pharmacogenetic study. Journal of Clinical Endocrinology & Metabolism.

[53] Zitzmann  M, Depenbusch  M (2004). X-chromosome inactivation patterns and androgen receptor functionality influence phenotype and social characteristics as well as pharmacogenetics of testosterone therapy in Klinefelter patients. Journal of Clinical Endocrinology & Metabolism.

[54] Zinn  AR, Ramos  P (2005). Androgen receptor CAG(n) repeat length influences phenotype of 47,XXY (Klinefelter) syndrome. Journal of Clinical Endocrinology & Metabolism.

[55] Stanford  JL, Just  JJ (1997). Polymorphic repeats in the androgen receptor gene: Molecular markers of prostate cancer risk. Cancer Research.

[56] Kantoff  P, Giovannucci  E (1998). The androgen receptor CAG repeat polymorphism and its relationship to prostate cancer. Biochimica et Biophysica Acta-Reviews on Cancer.

[57] Sartor  O, Zheng  Q (1999). Androgen receptor gene CAG repeat length varies in a race-specific fashion in men without prostate cancer. Urology.

[58] Hakimi  JM, Schoenberg  MP (1997). Androgen receptor variants with short glutamine or glycine repeats may identify unique subpopulations of men with prostate cancer. Clinical Cancer Research.

[59] Bratt  O, Borg  A (1999). CAG repeat length in the androgen receptor gene is related to age at diagnosis of prostate cancer and response to endocrine therapy, but not to prostate cancer risk. British Journal of Cancer.

[60] Ibanez  L, Ong  KK (2003). Androgen receptor gene CAG repeat polymorphism in the development of ovarian hyperandrogenism. Journal of Clinical Endocrinology & Metabolism.

[61] Mifsud  A, Ramirez  S (2000). Androgen receptor gene CAG trinucleotide repeats in anovulatory infertility and polycystic ovaries. Journal of Clinical Endocrinology & Metabolism.

[62] Hickey  T, Chandy  A (2002). The androgen-receptor CAG repeat polymorphism and X-chromosome inactivation in Australian Caucasian women with infertility related to polycystic ovary syndrome. Journal of Clinical Endocrinology & Metabolism.

[63] Kim  JJ, Choung  SH (2008). Androgen receptor gone CAG repeat polymorphism in women with polycystic ovary syndrome. Fertility and Sterility.

[64] Liu  QR, Hong  J (2008). Androgen receptor gene CAG(n) trinucleotide repeats polymorphism in Chinese women with polycystic ovary syndrome. Endocrine.

[65] Dasgupta  S, Sirisha  PVS (2010). Androgen Receptor CAG Repeat Polymorphism and Epigenetic Influence among the South Indian Women with Polycystic Ovary Syndrome. Plos One.

[66] Laisk  T, Haller-Kikkatalo K (2010). Androgen receptor epigenetic variations influence early follicular phase gonadotropin levels. Acta Obstetricia et Gynecologica Scandinavica.

[67] Radian  S, Baculescu  N (2010). CAG repeat alleles of the androgen receptor are associated with polycystic ovary syndrome (PCOS) in the Romanian population. Endocrine Abstracts.

[68] Skrgatic  L, Baldani  DP (2012). CAG repeat polymorphism in androgen receptor gene is not directly associated with polycystic ovary syndrome but influences serum testosterone levels. Journal of Steroid Biochemistry and Molecular Biology.

[69] Wang  R, Goodarzi  MO (2012). Negative association between androgen receptor gene CAG repeat polymorphism and polycystic ovary syndrome? A systematic review and meta-analysis. Molecular Human Reproduction.

[70] Mohlig  M, Jurgens  A (2006). The androgen receptor CAG repeat modifies the impact of testosterone on insulin resistance in women with polycystic ovary syndrome. European Journal of Endocrinology.

[71] Van Nieuwerburgh F, Stoop  D (2008). Shorter CAG repeats in the androgen receptor gene may enhance hyperandrogenicity in polycystic ovary syndrome. Gynecological Endocrinology.

[72] Heard  E, Clerc  P (1997). X-chromosome inactivation in mammals. Annual Review of Genetics.

[73] Eisner  JR, Dumesic  DA (2003). Increased adiposity in female rhesus monkeys exposed to androgen excess during early gestation. Obesity Research.

[74] Xu  N, Kwon  S (2011). Epigenetic Mechanism Underlying the Development of Polycystic Ovary Syndrome (PCOS)-Like Phenotypes in Prenatally Androgenized Rhesus Monkeys. Plos One.

[75] Nestler  JE (1987). Modulation of aromatase and P450 cholesterol side-chain cleavage enzyme activities of human placental cytotrophoblasts by insulin and insulin-like growth factor I. Endocrinology.

[76] Nestler  JE (1989). Insulin and insulin-like growth factor-I stimulate the 3 betahydroxysteroid dehydrogenase activity of human placental cytotrophoblasts. Endocrinology.

[77] Barbieri  RL, Saltzman  DH (1986). Elevated concentrations of the beta-subunit of human chorionic gonadotropin and testosterone in the amniotic fluid of gestations of diabetic mothers. Am J Obstet Gynecol.

[78] Zhu  JQ, Zhu  L (2010). Demethylation of LHR in dehydroepiandrosterone-induced mouse model of polycystic ovary syndrome. Molecular Human Reproduction.

[79] Giwercman  YL, Xu  C (1998). No association between the androgen receptor gene CAG repeat and impaired sperm production in Swedish men. Clinical Genetics.

[80] Edwards  A (1992). Genetic variation at five trimeric and tetrameric tandem repeat loci in four human population groups. Genomics.

[81] Lange  EM, Sarma  AV (2008). The androgen receptor CAG and GGN repeat polymorphisms and prostate cancer susceptibility in African-American men: results from the Flint Men's Health Study. Journal of Human Genetics.

[82] Vottero  A, Stratakis  CA (1999). Androgen receptor-mediated hypersensitivity to androgens in women with nonhyperandrogenic hirsutism: Skewing of X-chromosome inactivation. Journal of Clinical Endocrinology & Metabolism.

[83] Calvo  RM, Asuncion  M (2000). The role of the CAG repeat polymorphism in the androgen receptor gene and of skewed X-chromosome inactivation, in the pathogenesis of hirsutism. Journal of Clinical Endocrinology & Metabolism.

[84] Xu  N, Azziz  R (2010). Epigenetics in polycystic ovary syndrome: a pilot study of global DNA methylation. Fertility and Sterility.

[85] Heemers  HV, Tindall  DJ (2007). Androgen receptor (AR) coregulators: A diversity of functions converging on and regulating the AR transcriptional complex. Endocrine Reviews.

